# Optimizing Bioactive Profiles in Kolovi Olive Oils: Impact of Destoning, Harvest Timing, and Postharvest Factors on Phenolic, Tocopherol, Lutein, and Squalene Content

**DOI:** 10.3390/molecules31071181

**Published:** 2026-04-02

**Authors:** Ioannis C. Martakos, Ilias F. Tzavellas, Georgia Soultani, Nikolaos S. Thomaidis

**Affiliations:** Laboratory of Analytical Chemistry, Department of Chemistry, National and Kapodistrian University of Athens, Panepistimiopolis Zographou, 15771 Athens, Greece; johnmrtk@chem.uoa.gr (I.C.M.); tzavell@chem.uoa.gr (I.F.T.); gesoultan@chem.uoa.gr (G.S.)

**Keywords:** extra virgin olive oil (EVOO), Kolovi cultivar, phenolic compounds, tocopherols, squalene, processing parameters

## Abstract

Extra virgin olive oil (EVOO) is a key component of the Mediterranean diet, valued for its bioactive constituents and associated health benefits. This study evaluated the influence of four agronomic and processing factors—harvest month, destoning, fruit washing, and bottling delay—on the chemical composition of Kolovi EVOOs from the PGI Lesvos region. A total of 34 oils were produced under standardized conditions and analyzed for phenolic compounds, tocopherols, pigments, and squalene using UPLC-QTOF-MS and HPLC-DAD. The oils were characterized by consistently high nutritional quality, with most samples fulfilling EFSA health claim thresholds for hydroxytyrosol, tyrosol and its derivatives, and α-tocopherol. Harvest month was the most influential parameter: early harvested oils (October) contained significantly higher levels of phenolics, α-tocopherol, and lutein, whereas later harvests (November) were richer in squalene. Destoning produced modest changes, with slightly higher phenolics in non-destoned oils and reduced lipophilic antioxidants in destoned samples. Fruit washing selectively decreased hydrophilic phenolics, while lipophilic compounds were largely unaffected. Bottling delays of up to 48 h under protective conditions had negligible effects on composition, aside from minor increases in specific phenolic derivatives. These findings suggest that early harvesting and careful consideration of destoning are the most effective strategies for supporting the antioxidant profile of Kolovi EVOOs, while other practices can be adjusted with limited impact on quality.

## 1. Introduction

Extra virgin olive oil (EVOO) is the cornerstone of the Mediterranean diet and a subject of increasing scientific interest due to its rich chemical composition. Among the numerous bioactive compounds, phenolics such as secoiridoids, benzoic acids, cinnamic acids, flavonoids and lignans, tocopherols, carotenoid pigments (such as lutein and β-carotene), and squalene have been in the research spotlight in recent years [1,2]. These compounds contribute collectively to the nutritional profile and oxidative stability of EVOO and have been associated with various health benefits, including antioxidant, anti-inflammatory, and cardioprotective properties [3,4]. While polyphenols form the basis of the EFSA-authorized health claim (EU 432/2012) [5], the co-presence of lipophilic antioxidants, such as tocopherols and carotenoids, as well as other bioactive compounds, such as poly- and mono-unsaturated fatty acids, further enhance the oil’s functional potential and technological value [6].

Due to the large-scale production of EVOO in Mediterranean nations, there is a long-standing and increasing scientific focus on post-harvest and processing parameter optimization—specifically, malaxation conditions, storage procedures, and extraction methodologies [1,7,8]. A major limitation of existing research is the tendency to examine these parameters in isolation, rather than considering how they could interact. This reductionist approach overlooks the complex interplay between processing factors and the broader set of agronomic, biochemical, and technological influences that together shape EVOO quality and its bioactive profile.

In previous research by our laboratory, systematic analyses have been conducted on olive oils from several Greek olive cultivars such as Kolovi, Adramytiani, Koroneiki and more, including exploration of how different production practices, harvesting periods, olive fruit maturities, and several processing conditions influence both chemical, nutritional characteristics and health-protective properties of the resulting oil [1,9,10]. Such an approach points to the value of considering multiple parameters together, rather than treating each factor in isolation.

The present study focuses on *Olea europaea* L. cv. Kolovi, a cultivar predominantly grown on the island of Lesvos and designated as a Protected Geographical Indication (PGI) product. Previous analytical studies have shown that Kolovi oils are characterized by relatively rich phenolic profiles and notable concentrations of bioactive compounds, including phenolics, tocopherols, and pigments, when compared with typical ranges reported for extra virgin olive oils [1,9,10]. Reported values of total phenolics in Kolovi oils often exceed the threshold set by EFSA (250 mg/kg), highlighting the potential nutritional and functional importance of this cultivar. Despite this favourable compositional profile, the cultivar remains under-represented in international literature, particularly in the context of multifactorial process optimization. Previous studies on EVOO production have tended to assess agronomic and processing parameters in isolation, overlooking their interactions and thus failing to capture the complexity of factors that together shape oil composition and quality.

To address this gap, the present study evaluates the effect of four parameters—destoning, bottling delay, fruit washing, and harvest month—on the chemical and nutritional characteristics of Kolovi EVOOs. Each of these factors has been individually linked to compositional changes: bottling delay may influence enzymatic and oxidative pathways [11,12], destoning has been suggested to alter enzymatic profiles [13,14,15], and fruit washing can lead to reduced contaminants, cross-contamination, and has also been linked with decreases in positive sensory attributes via changes in phenolics [16,17]. Harvest month reflects fruit maturity and environmental influence on bioactive biosynthesis [18,19]. However, most of these variables have rarely been studied in parallel, leaving their combined impact insufficiently understood. By adopting a factorial design and applying targeted quantification techniques through UPLC-QTOF-MS and HPLC-DAD, this study provides a holistic assessment of how processing and agronomic factors affect the phenolic, tocopherol, pigment, and squalene content of Kolovi PGI EVOOs.

## 2. Results and Discussion

### 2.1. Nutritional Evaluation and Bioactive Profile

The study of the chemical profiles of the 34 Kolovi EVOO samples revealed substantial variation in both polar and lipophilic antioxidant constituents, reflecting the influence of agronomic and technological factors. Overall, the oils demonstrated high nutritional value, with most samples achieving levels associated with recognized health benefits. Among the samples analyzed, 91% met the EFSA health claim threshold for hydroxytyrosol, tyrosol and its derivatives (secoiridoids) (≥250 mg/kg) (Table 1), while 100% exceeded the vitamin E nutritional claim (α-tocopherol ≥ 90 mg/kg, according to EU Regulation 1169/2012Annex XIII [20]). The total phenolic content (TPC) ranged from 54 to 469 mg/kg, highlighting the wide dynamic range achievable within a single cultivar when key production parameters are modulated. The phenolic fraction was dominated by secoiridoid derivatives, including oleocanthal, oleacein, and oleuropein aglycone, which are closely associated with the sensory intensity and biological functionality of EVOO [21,22]. Among the detected secoiridoid derivatives, oleomissional and oleokoronal, previously described in olive oil by Diamantakos et al. [23], were also identified in the analyzed samples. Although a large proportion of the samples exceeded the EFSA thresholds for hydroxytyrosol and its derivatives and α-tocopherol, these results should not be interpreted as implying a commercial health claim but rather as a compositional benchmark within the studied dataset.

α-tocopherol concentrations varied between 168 and 318 mg/kg (Table 2), further reinforcing the oils’ antioxidant profile. These values are considered high relative to reported averages for Mediterranean cultivars and confirm the potential of Kolovi as a cultivar capable of delivering both oxidative stability and nutritional enhancement [1,9,24]. In addition to tocopherols, squalene concentrations ranged from 1661 to 2920 mg/kg. As a lipophilic triterpene with recognized bioactivity and skin-protective properties, squalene contributes to the oil’s health-promoting potential and market differentiation [25,26].

Pigment analysis showed lutein levels ranging from 0.26 to 0.96 mg/kg. Lutein, along with other carotenoids, not only contributes to the visual appearance of the oil by giving a yellow-gold colour but also enhances its antioxidant capacity through singlet oxygen quenching and potential synergism with polyphenols and tocopherols [27].

Taken together, these compositional results validate the Kolovi cultivar’s inherent capacity to yield EVOO with elevated concentrations of health-related compounds. The marked variability observed across samples also underscores the sensitivity of these compounds to even moderate shifts in agronomic or processing conditions.

### 2.2. Effect of Agronomic and Processing Parameters

ANOVA revealed that harvest month was the dominant factor shaping the compositional profile of Kolovi EVOOs, significantly affecting a broad spectrum of phenolic compounds, tocopherols, and squalene concentrations. Oils produced in November generally exhibited higher concentrations of squalene and simple phenolics such as tyrosol, while October oils were characterized by elevated levels of secoiridoid derivatives, reflecting known biochemical transformations during ripening. Destoning, although less influential than harvest timing, exerted consistent effects on key secoiridoids, with non-destoned samples retaining higher concentrations of oleocanthal, oleacein, and α-tocopherol in line with the proposed enzymatic and protective roles of the olive pit. Fruit washing was associated with selective changes, particularly reducing levels of luteolin, apigenin, and eriodictyol, pointing to partial removal of surface-bound phenolics despite its technological importance for cleanliness. Bottling delay (0, 24, or 48 h after centrifugation) showed only modest effects, with slight increases in oleomissional and vanillin in delayed samples, but no significant differences in total phenolic content, tocopherols, or pigments.

Importantly, several two-way interactions were detected, underscoring the multifactorial nature of EVOO composition. A comprehensive summary of the significant main and interaction effects identified by single-factor and multifactor ANOVA is provided in Table S2 of the ESM. For instance, total phenolics were jointly influenced by the combination of washing and bottling delay, suggesting that washing may alter the stability of phenolics during subsequent bottling steps. Tyrosol levels showed an interaction between harvest month and bottling delay, with bottling effects being more pronounced in November oils. Similarly, syringaresinol responded to a destoning × bottling interaction, while eriodictyol and naringenin exhibited harvest month × washing interactions, indicating that the impact of washing on flavonoids depends on fruit maturity stage. Collectively, these findings highlight that while harvest month is the primary driver of compositional variability, processing steps such as destoning, washing, and bottling timing not only introduce compound-specific modulations but also interact in ways that may amplify or mitigate their individual effects.

**Figure 1:**
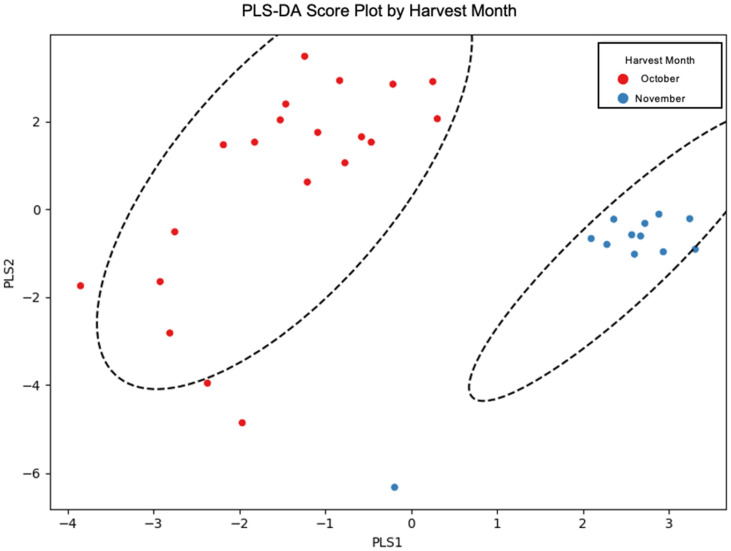
Partial least squares discriminant analysis (PLS-DA) score plot of Kolovi EVOO samples harvested in October (red) and November (blue). Clear separation between the two groups indicates distinct chemical profiles associated with harvest month. The ellipses represent 95% confidence intervals for each group. Model validation statistics are summarized in Table S3, confirming the robustness and predictive ability of the PLS-DA model.

Harvest maturity emerged as the most influential variable across all classes of bioactive compounds. Oils produced from October-harvested olives exhibited significantly higher levels of total phenolics, oleuropein aglycone, α-tocopherol, and lutein compared to those extracted in November. The mean increase in total phenolic content was approximately 10%, while α-tocopherol presented a 20% difference in the average concentrations. Lutein concentrations also followed the same trend but with higher decreases from October to November (almost 70%). This outcome reflects the biosynthetic peak of antioxidant compounds during early ripening stages and their subsequent degradation with fruit maturation. In contrast, squalene concentrations were generally higher in November oils, consistent with its accumulation during advanced ripening phases [28]. To further explore the influence of harvest month on the chemical composition of Kolovi EVOOs, partial least squares discriminant analysis (PLS-DA) was performed (Figure 1).

**Figure 2:**
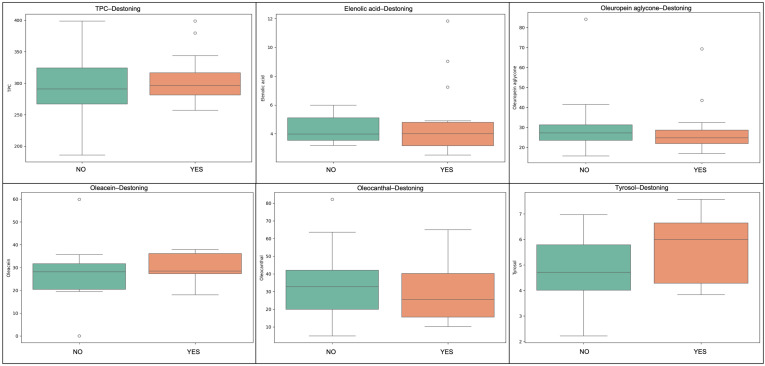
Boxplots showing the effect of destoning on selected phenolic and antioxidant compounds in Kolovi EVOOs. Parameters include total phenolic content (TPC), elenolic acid, oleuropein aglycone, oleacein, oleocanthal, and tyrosol as examples.

**Figure 3:**
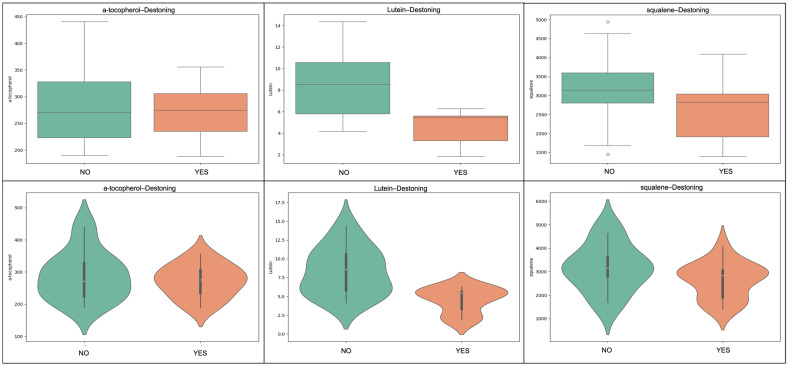
The influence of destoning on lipophilic bioactives in Kolovi EVOOs is illustrated. Boxplots (top row) and violin plots (bottom row) compare α-tocopherol, lutein, and squalene concentrations in oils obtained from non-destoned (NO) versus destoned (YES) olives. Destoned samples generally exhibited reduced levels of these compounds, suggesting a cultivar–dependent impact of pit removal on lipophilic antioxidant retention. The width of each violin represents the probability density of the data. The embedded boxplots indicate the interquartile range (IQR), with the central line representing the median value and whiskers extending to 1.5× IQR.

The destoning process did not significantly affect the compositional profile of the oils; however, some consistent trends were observed. Samples produced from non-destoned olives exhibited higher levels of total phenolics, with an approximately 3% increase in the mean values. In contrast, destoned samples showed a reduction in lipophilic antioxidants such as α-tocopherol, lutein and squalene. Although these differences were modest, the observed trend is chemically plausible and may be related to the role of the olive pit during the processing of olives and production of olive oil. The presence of the pit influences the mechanical structure of the olive paste during crushing and malaxation and may affect the activity of endogenous enzymes responsible for the transformation of phenolic compounds. In addition, the pit may influence oxygen diffusion and microstructural characteristics of the paste, which can impact oxidative processes and the stability of lipophilic antioxidants. Previous studies investigating destoned olive oils have reported variable outcomes depending on processing conditions and cultivar characteristics [13,15]. Frangipane et al. [15] highlighted that the effect of destoning on phenolic compounds can vary significantly among cultivars, with some varieties showing increases in secoiridoid derivatives while others exhibit more limited changes. These observations suggest that the impact of destoning may be cultivar-dependent, and the response observed in Kolovi oils may reflect specific biochemical or structural characteristics of this cultivar. Therefore, while destoning is often considered for technological or sensory optimization, its influence on antioxidant retention should be evaluated carefully on a cultivar-specific basis. In Figure 2 and Figure 3, box and violin plots for selected compounds are presented.

**Figure 4:**
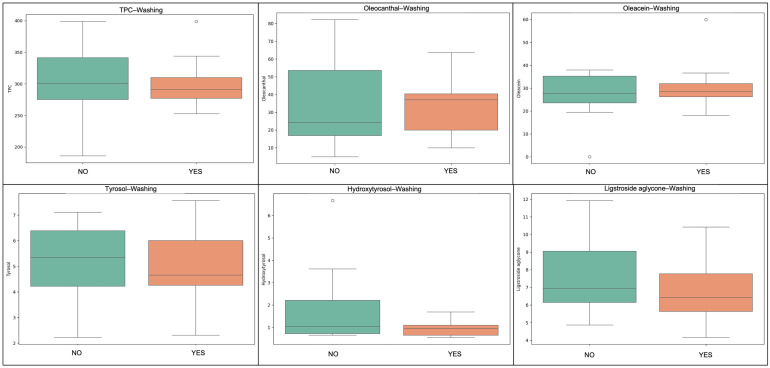
The effect of fruit washing prior to malaxation on selected phenolic compounds in Kolovi EVOOs is presented. Boxplots compare washed (YES) vs. unwashed (NO) samples for total phenolic content (TPC), oleocanthal, oleacein, tyrosol, hydroxytyrosol, and ligstroside aglycone. Washed samples generally exhibited lower phenolic levels, suggesting a partial removal or dilution of hydrophilic antioxidants during the washing step.

**Figure 5:**
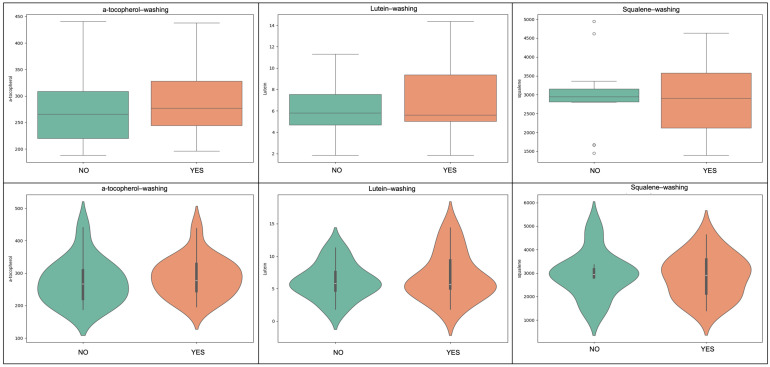
The effect of fruit washing on lipophilic constituents of Kolovi EVOOs is shown. Boxplots (top row) and violin plots (bottom row) compare washed (YES) versus unwashed (NO) samples for α-tocopherol, lutein, and squalene. The distributions indicate that fruit washing exerts compound-specific effects, with variable impacts on antioxidant and pigment concentrations. The width of each violin represents the probability density of the data. The embedded boxplots indicate the interquartile range (IQR), with the central line representing the median value and whiskers extending to 1.5× IQR.

Washing of the fruit prior to oil production resulted in moderate reductions in hydrophilic phenolics. Compounds such as ligstroside aglycone and tyrosol were particularly affected, while lipophilic constituents (tocopherols, squalene, pigments) remained largely unaffected. The results align with the hypothesis that water-soluble antioxidants are vulnerable to loss during aqueous contact, especially in cases of prolonged or vigorous washing [16,17]. As illustrated in Figure 4 and Figure 5, washed samples generally exhibited slightly lower concentrations of several hydrophilic phenolic compounds, whereas the distributions of lipophilic compounds such as α-tocopherol, lutein, and squalene remained relatively similar between washed and non-washed samples. Despite its necessity in specific sanitary contexts, washing introduces a minor nutritional cost that should be considered in premium EVOO production.

**Figure 6:**
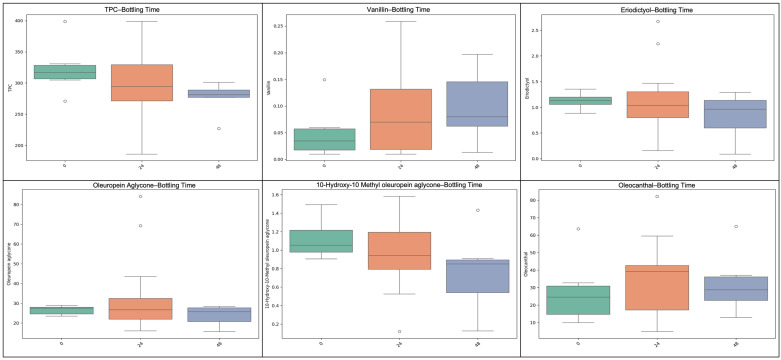
The effect of bottling delay (0 h, 24 h, 48 h) on selected phenolic compounds of Kolovi EVOOs is presented. Boxplots compare total phenolic content (TPC), vanillin, elenolic acid, oleuropein aglycone, 10-hydroxy-(10-methyl) oleuropein aglycone, and oleocanthal across the three bottling delay levels.

**Figure 7:**
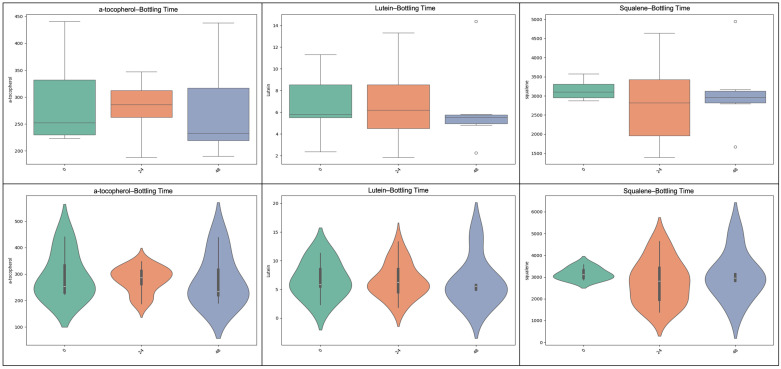
Impact of bottling delay (0 h, 24 h, 48 h) on lipophilic antioxidants and pigments of Kolovi EVOOs is shown. Boxplots (top row) and violin plots (bottom row) illustrate the distributions of α-tocopherol, lutein, and squalene concentrations, highlighting compound-specific responses to delayed bottling. The width of each violin represents the probability density of the data. The embedded boxplots indicate the interquartile range (IQR), with the central line representing the median value and whiskers extending to 1.5× IQR.

Bottling delays of 0, 24, and 48 h after centrifugation influenced the chemical profiles of Kolovi EVOOs in a compound-specific manner (Figure 6 and Figure 7). Among the phenolic derivatives, oleocanthal and vanillin increased progressively with longer delays. This trend suggests that residual enzymatic activity remains active for a short period after oil production and separation, converting secoiridoid precursors into downstream products. By contrast, total phenolic content and the main aglycones (oleuropein aglycone and hydroxy-methyl oleuropein aglycone) remained unchanged, pointing to limited oxidative degradation under the conditions applied.

Regarding the lipophilic antioxidants, concentrations of α-tocopherol, lutein, and squalene were stable across all bottling delay levels. Their preservation underscores the effectiveness of cold storage and oxygen-free handling in protecting tocopherols and carotenoids, compounds that are otherwise highly susceptible to oxidative loss. These outcomes differ from previous reports in which delayed handling under non-controlled conditions accelerated the degradation of pigments and tocopherols [29,30], highlighting the importance of a controlled bottling environment.

The data obtained by this study suggest that short-term bottling delays, when carried out under protective conditions, do not compromise the stability of key lipophilic antioxidants. Moreover, such delays may even allow subtle adjustments in phenolic composition, potentially enhancing the oil’s profile without sacrificing quality.

## 3. Materials and Methods

### 3.1. Chemicals and Standards

For the determination of phenolic compounds, methanol (MeOH) and 2-propanol of LC-MS grade were obtained from Merck (Darmstadt, Germany). Ultrapure water was produced in our laboratory with the use of a Milli-Q Direct-Q UV purification system (Millipore, Bedford, MA, USA). Sodium hydroxide monohydrate for trace analysis ≥ 99.9995% and ammonium acetate ≥ 99.0% were obtained from Fluka (Buchs, Switzerland).

Commercially available standards were obtained from the following suppliers: hydroxytyrosol and luteolin from Santa Cruz Biotechnology (Santa Cruz, CA, USA); apigenin, naringenin, tyrosol, and vanillin from Alfa Aesar (Karlsruhe, Germany); and p-coumaric acid, eriodictyol, pinoresinol, and syringaldehyde from Sigma-Aldrich (Steinheim, Germany). Additional compounds—including ligstroside aglycone, oleacein, oleocanthal, oleocanthalic acid, oleomissional, and oleuropein aglycone—were provided by the Laboratory of Pharmacognosy and Natural Products Chemistry, Faculty of Pharmacy, University of Athens (Athens, Greece). These were previously isolated from olive oil extracts, and their identity and purity were confirmed by nuclear magnetic resonance (NMR) spectroscopy [23].

For the analysis of tocopherols, pigments, and squalene, MeOH and acetonitrile (ACN) of HPLC grade were purchased from Fisher Scientific (Geel, Belgium), while IPA was obtained from Honeywell (Offenbach, Germany). Standard compounds—lutein, α-, γ-, and δ-tocopherol, and squalene—were acquired from Sigma-Aldrich (Steinheim, Germany). Stock solutions were prepared as described thoroughly by Martakos et al. [1].

### 3.2. Sample Collection and Experimental Design

A total of 34 EVOO samples derived from the Kolovi cultivar were collected during the 2024–2025 crop year on the island of Lesvos (PGI region). The study applied a factorial design integrating four parameters: (i) destoning (Yes/No); (ii) bottling delay (0 h, 24 h, 48 h after production); (iii) fruit washing (Yes/No); (iv) harvest month (October/November). The allocation was partially balanced overall (Washing: 17/17; Destoning: 15/19) but unbalanced for Month (October = 21; November = 13) and Bottling delay (0 h = 6; 24 h = 20; 48 h = 8). Table 3 summarizes the full sample metadata. All samples were obtained through pilot-scale extraction under standardized malaxation conditions (25 °C for 45 min) and stored in amber glass vials at 4 °C prior to analysis. The fruit matrix was standardized to Kolovi-rich blends (≥75%) to ensure comparability across conditions.

### 3.3. Phenolic Compound Analysis

#### 3.3.1. Sample Preparation

An aliquot of 0.50 g of olive oil was weighed in an Eppendorf tube and spiked with 100 μL of internal standard (syringaldehyde, 10 mg L^−1^). For the extraction, 400 μL of a mixture containing methanol/water (80:20, *v*/*v*) was used. The mixture was vortexed for 1 min. Following phase separation, the upper (polar) phase was transferred to a 2 mL vial and filtered through 0.2 μm regenerated cellulose (RC) syringe filters supplied by Macherey-Nagel (Düren, Germany). The analytical methodology has been validated in a study previously published by our laboratory [8]. Therefore, single analytical measurements were considered sufficient for the purposes of comparative phenolic profiling across samples.

#### 3.3.2. Instrumental Conditions

Instrumental analysis was performed on an ultra-high-performance chromatography (UPLC) system (Dionex UltiMate 3000 RSLC, Thermo Fisher Scientific, Dreieich, Germany) coupled to a QTOF-MS (Maxis Impact, Bruker Daltonics, Bremen, Germany) with electrospray ionization (ESI) in negative ionization mode. Chromatographic separation was achieved using an Acclaim RSLC 120 C18 column (2.2 μm, 2.1 × 100 mm, Thermo Fisher Scientific, Dreieich, Germany) kept constantly at 30 °C. Mobile phases A and B consisted of: (A) 10 mM ammonium acetate in water/MeOH (90:10) and (B) 10 mM ammonium acetate in MeOH, with a gradient elution at 0.2–0.48 mL min^−1^. Mass range was set at *m*/*z* 50–1000. External calibration was performed using a sodium formate solution, injected prior to each chromatographic run in a calibration segment from 0.1 to 0.25 min. Phenolic compounds were identified via an in-house database of 96 known olive phenolics, based on retention time (ΔtR ≤ 0.2 min), mass accuracy (<2 mDa), isotopic fit (<50 mSigma), and fragmentation pattern (≥2 confirmatory ions).

### 3.4. Tocopherol, Pigment, and Squalene Analysis

#### 3.4.1. Sample Preparation

For pigment, tocopherol and squalene analysis, 0.1 g of oil was accurately weighed into an Eppendorf tube supplied by Eppendorf SE (Hamburg, Germany), followed by the addition of 900 μL of IPA. The mixture was vortexed for 1 min, filtered through the same type of RC filters and transferred to a 2 mL glass vial. Finally, 20 μL were injected into the HPLC system.

#### 3.4.2. Instrumental Conditions

Analyses were conducted on an HPLC system (Shimadzu LC-2030C 3D Plus, by Shimadzu Corporation, Kyoto, Japan) in reversed-phase mode with the use of a Spherisorb ODS2 C18 column (5 μm, 4.6 × 250 mm, Waters, Milford, MA, USA). Mobile phases were methanol (A) and acetonitrile (B), running in a gradient elution programme. Detection was performed at 295 nm (tocopherols), 450 nm (lutein,), and 210 nm (squalene). Identification was based on retention time and spectral matching with standard curves. The method has been validated by our laboratory, as described in a previous publication [1].

### 3.5. Statistical Analysis

Statistical analyses were performed using Python (version 3.11), with multifactor ANOVA conducted using statsmodels (version 0.14) and data processing carried out using pandas (version 2.1) and NumPy (version 1.26). Quantitative data were analyzed using multifactor ANOVA, evaluating the significance of agronomic and processing parameters (harvest month, destoning, fruit washing, and bottling delay) on individual phenolic compounds, tocopherols, pigments, and total phenolics. Because the experimental dataset was not fully balanced across all factor combinations, multifactor ANOVA was performed using Type II sums of squares, allowing estimation of the main effects of harvest month, destoning, washing, and bottling delay while accounting for unequal sample sizes between factor levels [31]. In parallel, multivariate analyses including PLS-DA were performed to assess sample clustering and variable contributions based on the full compositional dataset.

## 4. Conclusions

This study shows that the Kolovi cultivar has the capacity to produce EVOO consistently rich in phenolics, tocopherols, and squalene, with most samples meeting established health claim thresholds for tocopherols and hydroxytyrosol and its derivatives. Among the factors investigated, harvest month and destoning had the most pronounced effects on the bioactive profile. Early harvesting (October) was associated with higher levels of phenolics, α-tocopherol, and lutein, whereas later harvests favoured squalene accumulation. Non-destoned olives tended to yield slightly higher phenolic concentrations, while destoning was linked to lower levels of lipophilic antioxidants. Fruit washing selectively reduced hydrophilic phenolics, while lipophilic constituents remained largely unaffected. Bottling delays of up to 48 h, when carried out under protective conditions, had minimal impact on oil composition apart from small increases in certain phenolic derivatives.

Taken together, these results highlight the importance of considering multiple agronomic and technological decisions in a coordinated manner. For Kolovi EVOO, early harvesting and careful evaluation of destoning appear to be the most effective strategies for supporting nutritional quality, while other practices can be adjusted according to operational needs with limited risk to overall composition. These findings provide a basis for informed production choices in the PGI Lesvos region and contribute to a broader understanding of how processing and agronomic factors shape EVOO quality.

## Figures and Tables

**Table 1:** Concentrations of selected phenolic compounds detected in Kolovi extra virgin olive oil samples (mg/Kg). Values correspond to single analytical measurements obtained by UPLC-QTOF-MS. Statistical evaluation of treatment effects is presented separately in Table S2 (Electronic Supplementary Material).

Analyte/Sample	Hydroxytyrosol	Luteolin	Oleacein	Oleocanthal	Oleokoronal	Oleomissional	Oleuropein Aglycone	Tyrosol	Total Phenolic Content
Sample 1	1.21	2.08	20.1	22.2	33.7	101	15.7	4.61	227
Sample 2	6.68	1.98	0.3	4.9	40.0	96.8	16.0	2.22	186
Sample 3	1.33	6.97	36.5	40.1	57.2	96.6	32.4	4.35	297
Sample 4	1.44	7.91	31.9	37.0	55.8	88.7	27.8	4.38	277
Sample 5	1.69	7.36	28.1	44.3	66.0	103	32.7	4.01	309
Sample 6	3.62	1.40	37.9	51.4	110	86.8	69.3	3.84	399
Sample 7	3.07	1.85	20.5	82.2	55.8	77.4	84.2	2.24	352
Sample 8	0.98	7.25	18.1	40.4	96.2	113	43.5	4.06	344
Sample 9	3.37	1.09	36.9	65.0	42.6	82.1	28.5	6.90	301
Sample 10	0.76	7.91	19.5	31.4	40.0	76.7	31.3	4.72	231
Sample 11	0.90	7.26	20.4	42.1	96.0	104	26.3	5.18	321
Sample 12	0.84	7.49	20.7	38.5	77.9	84.4	24.8	4.32	277
Sample 13	1.10	3.45	59.9	41.9	38.6	77.3	27.3	2.31	291
Sample 14	0.96	6.87	23.8	33.2	76.7	95.1	27.4	4.26	285
Sample 15	1.10	6.27	26.7	39.8	75.9	106	32.1	4.95	310
Sample 16	1.02	7.00	26.6	32.7	90.9	122	27.2	5.35	399
Sample 17	1.03	5.90	26.2	63.6	84.8	164	28.0	6.31	380
Sample 18	1.20	6.97	27.5	55.7	83.1	155	23.3	7.11	322
Sample 19	1.03	8.28	27.1	23.6	68.7	139	27.9	6.40	305
Sample 20	1.06	7.06	28.4	25.6	74.5	112	28.9	7.12	410
Sample 21	0.91	6.04	36.5	60.5	58.8	175	51.6	3.00	285
Sample 22	0.80	6.33	29.3	24.1	68.0	113	23.9	6.67	290
Sample 23	1.35	4.12	32.7	59.5	49.5	95.6	41.5	3.34	325
Sample 24	0.59	6.72	36.6	23.0	65.4	111	17.0	7.57	285
Sample 25	0.65	5.75	31.7	19.9	52.5	97.4	19.5	6.98	253
Sample 26	0.66	4.95	34.8	16.2	58.1	112	22.0	6.00	273
Sample 27	0.68	3.99	35.7	17.5	88.4	158	29.9	5.79	358
Sample 28	0.79	4.37	35.0	15.0	62.8	93.4	21.7	6.01	257
Sample 29	0.65	4.41	35.8	12.9	71.9	110	19.7	6.39	278
Sample 30	0.62	4.04	29.9	11.4	62.8	114	22.8	5.79	267
Sample 31	0.63	3.45	30.2	9.9	50.7	133	23.5	4.66	271
Sample 32	0.66	3.16	27.8	10.2	54.0	156	20.8	4.84	292
Sample 33	0.55	3.73	28.2	11.7	60.6	166	23.7	3.94	312
Sample 34	0.62	3.33	30.4	10.9	50.1	153	26.5	3.19	293

**Table 2:** Concentrations of lipophilic compounds measured in Kolovi EVOO samples. Values represent single analytical measurements obtained by HPLC-DAD and are expressed in mg/kg of olive oil. Statistical evaluation of treatment effects was performed using multifactor ANOVA and is reported separately in Table S2 (Electronic Supplementary Material).

Sample/Analyte	α-Tocopherol	(β + γ)-Tocopherol	Squalene	Lutein
Sample 1	190	8.4	3168	5.8
Sample 2	307	16	3026	10.6
Sample 3	277	17	2805	5.5
Sample 4	438	12	2909	14.4
Sample 5	333	24	2127	10.4
Sample 6	316	27	1676	6.2
Sample 7	271	10	2803	8.5
Sample 8	295	25	1831	5.6
Sample 9	221	8.0	3008	4.8
Sample 10	252	29	1450	8.6
Sample 11	318	33	1681	10.7
Sample 12	275	27	1999	6.2
Sample 13	269	25	2124	5.0
Sample 14	341	12	2792	5.5
Sample 15	347	26	3754	13.3
Sample 16	441	13	2872	11.3
Sample 17	223	10	3362	9.4
Sample 18	266	11	3364	6.3
Sample 19	356	13	2914	5.6
Sample 20	225	8.4	3135	5.5
Sample 21	231	8.4	3446	5.7
Sample 22	219	21	4948	5.6
Sample 23	208	11	4616	4.6
Sample 24	196	10	4093	1.8
Sample 25	328	48	4636	6.4
Sample 26	306	43	2828	4.3
Sample 27	311	49	3146	6.6
Sample 28	306	42	1394	4.3
Sample 29	245	16	1666	2.2
Sample 30	216	11	3602	4.2
Sample 31	244	21	3578	6.1
Sample 32	188	9.8	2949	1.8
Sample 33	261	18	3076	2.4
Sample 34	191	6.0	3076	8.4

**Table 3:** Sample metadata table.

Sample	Harvest Month	Washing	Destoning	Bottling Delay
1	October	No	No	48
2	October	No	No	24
3	October	Yes	Yes	24
4	October	Yes	No	48
5	October	Yes	No	24
6	October	No	Yes	24
7	October	No	No	24
8	October	Yes	Yes	24
9	October	No	Yes	48
10	October	No	No	24
11	October	Yes	No	24
12	October	Yes	Yes	24
13	October	Yes	No	24
14	October	Yes	Yes	48
15	October	Yes	No	24
16	October	No	No	0
17	October	Yes	No	0
18	October	No	Yes	24
19	October	No	Yes	0
20	October	Yes	Yes	0
21	October	No	No	48
22	November	No	No	48
23	November	No	No	24
24	November	Yes	Yes	24
25	November	Yes	No	24
26	November	No	Yes	24
27	November	No	No	24
28	November	Yes	Yes	24
29	November	No	Yes	48
30	November	Yes	No	24
31	November	Yes	No	0
32	November	No	Yes	24
33	November	Yes	Yes	0
34	November	No	No	48

## Data Availability

Dataset available on request from the authors.

## Supplementary Materials

The following supporting information can be downloaded at: https://www.mdpi.com/article/10.3390/molecules31071181/s1, Table S1. Phenolic compounds results expressed in mg/Kg; Table S2. Significant effects identified by single-factor and multifactor ANOVA for phenolic compounds and total phenolics in Kolovi EVOOs; Table S3. Validation metrics of the PLS-DA model used to discriminate olive oils according to harvest month. Model performance was evaluated using stratified 5-fold cross-validation. The optimal number of latent variables was selected based on the maximum Q^2^ value, and model robustness was assessed using permutation testing (n = 500).

## Abbreviations

The following abbreviations are used in this manuscript:

EVOO	Extra Virgin Olive Oil
PGI	Protected Geographical Indication
EFSA	European Food Safety Authority
EU	European Union
TPC	Total Phenolic Content
UPLC	Ultra-Performance Liquid Chromatography
QTOF-MS	Quadrupole Time-of-Flight Mass Spectrometry
HPLC	High-Performance Liquid Chromatography
DAD	Diode Array Detector
LC-MS	Liquid Chromatography–Mass Spectrometry
ESI	Electrospray Ionization
PLS-DA	Partial Least Squares Discriminant Analysis
MeOH	Methanol
ACN	Acetonitrile
IPA	Isopropanol (2-Propanol)
NMR	Nuclear Magnetic Resonance
*m*/*z*	Mass-to-Charge Ratio
ΔtR	Retention Time Deviation
ANOVA	Analysis of Variance
