# Supplementary material for: Optimizing Bioactive Profiles in Kolovi Olive Oils: Impact of Destoning, Harvest Timing, and Postharvest Factors on Phenolic, Tocopherol, Lutein, and Squalene Content
Source: Molecules. 2026 Apr 2;31(7):1181. doi: 10.3390/molecules31071181 (PMC13074956; doi:10.3390/molecules31071181)
Supplement: Supplementary file 1 [file molecules-31-01181-s001.zip › molecules-4159547-supplementary.pdf]

Optimising Bioactive Profiles in Kolovi Olive Oils: Impact of Destoning, Harvest Timing, and Postharvest Factors on Phenolic, Tocopherol, Lutein and Squalene Content

Ioannis C. Martakos, Ilias F. Tzavellas, Georgia Soultani and Nikolaos S. Thomaidis*

Electronic Supplementary Material

Electronic Supplementary Material

Table of contents

Table S1. Phenolic compounds results expressed in mg/Kg.. 3

Table S1. (cont.) Phenolic compounds results expressed in mg/Kg 4

Table S2. Significant effects identified by single-factor and multifactor ANOVA for phenolic compounds and total phenolics in Kolovi EVOOs. 5

Table S3. Validation metrics of the PLS-DA model used to discriminate olive oils according to harvest month. Model performance was evaluated using stratified 5-fold cross-validation. The optimal number of latent variables was selected based on the maximum Q^2 value, and model robustness was assessed using permutation testing (n = 500). 5

Table S1. Phenolic compounds results expressed in mg/Kg

Analyte/Sample	Sample 1	Sample 2	Sample 3	Sample 4	Sample 5	Sample 6	Sample 7	Sample 8	Sample 9	Sample 10	Sample 11	Sample 12	Sample 13	Sample 14	Sample 15	Sample 16	Sample 17
10-Hydroxy decarboxymethyl oleuropein aglycone	0.10	0.07	0.13	0.15	0.17	0.85	0.98	0.22	1.05	0.14	0.12	0.14	1.78	0.13	0.12	0.10	0.09
10-Hydroxy-10-Methyl oleuropein aglycone	0.13	0.12	0.77	0.84	0.78	0.91	0.53	0.95	0.44	0.80	0.94	0.94	0.68	0.91	1.04	0.96	1.07
10-Hydroxyoleuropein aglycone	0.01	0.02	0.08	0.08	0.07	0.25	0.09	0.06	0.09	0.05	0.06	0.06	0.11	0.05	0.04	0.05	0.06
1-Acetoxypinoresinol	1.07	0.89	1.85	1.60	1.73	1.41	2.16	1.58	3.87	1.35	1.57	1.76	5.67	1.64	1.86	2.20	2.62
Apigenin	0.41	0.91	2.10	1.82	1.79	0.89	1.72	2.00	1.40	1.51	1.43	1.52	2.69	1.32	1.20	1.24	1.36
Elenolic acid	5.42	3.54	7.25	5.98	5.11	11.84	3.95	4.89	9.03	5.39	4.34	4.69	5.52	4.01	3.40	3.58	3.39
Eriodictyol	0.09	0.16	0.80	0.92	0.93	0.50	0.49	0.78	0.49	0.80	0.94	1.05	2.24	1.01	1.09	1.07	0.89
Hydroxylated form of elenolic acid	1.13	0.25	0.10	0.10	0.08	0.21	0.00	0.07	0.05	0.08	0.08	0.09	0.10	0.09	0.09	0.08	0.09
Hydroxytyrosol	1.21	6.68	1.33	1.44	1.69	3.62	3.07	0.98	3.37	0.76	0.90	0.84	1.10	0.96	1.10	1.02	1.03
Hydroxytyrosol acetate	0.56	0.55	0.66	0.54	0.49	1.42	2.25	0.56	2.68	0.56	0.66	0.63	1.90	0.61	0.67	0.72	0.70
Lingstroside aglycone	11.93	5.37	6.42	7.65	8.20	11.58	10.97	7.77	9.09	5.89	6.72	5.64	10.42	5.59	6.27	6.90	8.00
Luteolin	2.08	1.98	6.97	7.91	7.36	1.40	1.85	7.25	1.09	7.91	7.26	7.49	3.45	6.87	6.27	7.00	5.90
Methyl oleuropein aglycone	0.13	0.04	0.16	0.14	0.14	0.05	0.09	0.15	0.08	0.19	0.18	0.15	0.19	0.12	0.10	0.11	0.11
Naringenin	0.08	0.05	0.14	0.12	0.12	0.12	0.13	0.14	0.11	0.14	0.16	0.16	0.30	0.13	0.14	0.12	0.10
Oleacein	20.1	0.0	36.5	31.9	28.1	37.9	20.5	18.1	36.9	19.5	20.4	20.7	59.9	23.8	26.7	26.6	26.2
Oleocanthal	22.2	4.9	40.1	37.0	44.3	51.4	82.2	40.4	65.0	31.4	42.1	38.5	41.9	33.2	39.8	32.7	63.6
Oleocanthalic acid	4.00	3.01	0.33	0.33	0.37	2.51	0.00	0.35	2.99	0.37	0.38	0.39	3.22	0.37	0.36	0.37	0.31
Oleokoronal	33.7	40.0	57.2	55.8	66.0	110	55.8	96.2	42.6	40.0	96.0	77.9	38.6	76.7	75.9	90.9	84.8
Oleomissional	101	96.8	96.6	88.7	103	86.8	77.4	113	82.1	76.7	104	84.4	77.3	95.1	106	122	164
Oleuropein aglycone	15.7	16.0	32.4	27.8	32.7	69.3	84.2	43.5	28.5	31.3	26.3	24.8	27.3	27.4	32.1	27.2	28.0
p-coumaric acid	0.80	0.61	0.50	0.47	0.39	0.18	0.06	0.26	0.25	0.36	0.38	0.32	0.38	0.30	0.34	0.29	0.34
Pinoresinol	0.67	0.78	0.55	0.53	0.47	1.75	1.03	0.55	1.91	0.56	0.67	0.68	3.37	0.62	0.60	0.66	0.63
Syringaresinol	0.07	0.36	0.03	0.15	0.09	0.20	0.47	0.11	0.58	0.38	0.45	0.02	0.56	0.01	0.43	0.02	0.09
Tyrosol	4.61	2.22	4.35	4.38	4.01	3.84	2.24	4.06	6.90	4.72	5.18	4.32	2.31	4.26	4.95	5.35	6.31
Vanillin	0.16	0.12	0.06	0.06	0.07	0.14	0.01	0.02	0.20	0.07	0.01	0.07	0.26	0.09	0.13	0.01	0.05
Phenolic Content	227	186	297	277	309	399	352	344	301	231	321	277	291	285	310	399	380

Table S1. (cont.) Phenolic compounds results expressed in mg/Kg

Analyte/Sample	Sample 18	Sample 19	Sample 20	Sample 21	Sample 22	Sample 23	Sample 24	Sample 25	Sample 26	Sample 27	Sample 28	Sample 29	Sample 30	Sample 31	Sample 32	Sample 33	Sample 34
10-Hydroxy decarboxymethyl oleuropein aglycone	0.09	0.09	0.08	0.07	0.06	1.35	0.05	0.06	0.06	0.06	0.06	0.07	0.06	0.05	0.06	0.06	0.06
10-Hydroxy-10-Methyl oleuropein aglycone	1.27	1.49	1.26	1.24	1.43	1.25	1.39	1.58	1.34	1.17	0.97	0.86	0.91	1.03	1.00	0.91	1.05
10-Hydroxyoleuropein aglycone	0.06	0.07	0.07	0.08	0.09	0.20	0.10	0.12	0.14	0.16	0.15	0.17	0.15	0.15	0.14	0.16	0.19
1-Acetoxypinoresinol	2.76	2.26	2.28	2.27	1.87	4.71	1.83	1.55	1.63	1.68	1.88	1.66	1.39	1.61	1.62	1.82	2.15
Apigenin	1.47	1.19	1.32	1.23	1.12	2.43	1.17	1.28	1.19	1.14	0.92	0.81	0.73	0.82	0.67	0.72	0.71
Elenolic acid	3.33	3.16	3.19	3.36	3.77	4.84	4.01	4.72	4.04	3.98	3.71	3.02	3.29	3.19	2.77	2.52	2.89
Eriodictyol	0.94	1.05	1.20	1.07	1.18	2.67	1.18	1.03	1.15	1.36	1.30	1.29	1.47	1.19	1.30	1.35	1.54
Hydroxylated form of elenolic acid	0.09	0.11	0.11	0.09	0.08	0.16	0.09	0.07	0.07	0.06	0.06	0.06	0.07	0.06	0.05	0.05	0.05
Hydroxytyrosol	1.20	1.03	1.06	0.91	0.80	1.35	0.59	0.65	0.66	0.68	0.79	0.65	0.62	0.63	0.66	0.55	0.62
Hydroxytyrosol acetate	0.57	0.59	0.68	0.58	0.55	2.21	0.46	0.47	0.54	0.54	0.51	0.57	0.61	0.64	0.57	0.67	0.55
Lingstroside aglycone	7.30	6.96	8.07	5.75	5.71	9.02	5.24	5.96	6.65	6.95	6.52	6.40	5.64	5.09	4.87	4.16	4.32
Luteolin	6.97	8.28	7.06	6.04	6.33	4.12	6.72	5.75	4.95	3.99	4.37	4.41	4.04	3.45	3.16	3.73	3.33
Methyl oleuropein aglycone	0.13	0.15	0.12	0.13	0.12	0.26	0.11	0.12	0.11	0.13	0.13	0.13	0.15	0.17	0.16	0.16	0.14
Naringenin	0.10	0.11	0.12	0.10	0.10	0.26	0.08	0.07	0.06	0.07	0.06	0.06	0.05	0.06	0.07	0.06	0.05
Oleacein	27.5	27.1	28.4	36.5	29.3	32.7	36.6	31.7	34.8	35.7	35.0	35.8	29.9	30.2	27.8	28.2	30.4
Oleocanthal	55.7	23.6	25.6	60.5	24.1	59.5	23.0	19.9	16.2	17.5	15.0	12.9	11.4	9.9	10.2	11.7	10.9
Oleocanthalic acid	0.27	0.25	0.22	0.22	0.20	2.71	0.19	0.21	0.22	0.26	0.23	0.20	0.16	0.15	0.15	0.16	0.17
Oleokoronal	83.1	68.7	74.5	58.8	68.0	49.5	65.4	52.5	58.1	88.4	62.8	71.9	62.8	50.7	54.0	60.6	50.1
Oleomissional	155	139	112	175	113	95.6	111	97.4	112	158	93.4	110	114	133	156	166	153
Oleuropein aglycone	23.3	27.9	28.9	51.6	23.9	41.5	17.0	19.5	22.0	29.9	21.7	19.7	22.8	23.5	20.8	23.7	26.5
p-coumaric acid	0.40	0.48	0.46	0.40	0.34	0.32	0.28	0.27	0.23	0.22	0.23	0.24	0.27	0.25	0.20	0.18	0.17
Pinoresinol	0.75	0.77	0.75	0.68	0.67	3.49	0.52	0.59	0.67	0.73	0.77	0.74	0.69	0.70	0.63	0.63	0.66
Syringaresinol	0.05	0.37	0.31	0.06	0.25	0.80	0.31	0.08	0.02	0.03	0.04	0.18	0.11	0.01	0.35	0.18	0.08
Tyrosol	7.11	6.40	7.12	3.00	6.67	3.34	7.57	6.98	6.00	5.79	6.01	6.39	5.79	4.66	4.84	3.94	3.19
Vanillin	0.03	0.02	0.06	0.03	0.01	0.23	0.02	0.04	0.01	0.02	0.11	0.07	0.18	0.02	0.16	0.15	0.02
Phenolic Content	322	305	410	285	290	325	285	253	273	358	257	278	267	271	292	312	293

Table S2. Significant effects identified by single-factor and multifactor ANOVA for phenolic compounds and total phenolics in Kolovi EVOOs.

Compound	Effect	df (effect, residual)	F-value	p-value
Apigenin	Harvest Month	1, 28	5,96	0.0212
Elenolic acid	Harvest Month	1, 28	5,83	0.0225
Eriodictyol	Harvest Month	1, 28	11,61	0.0020
Eriodictyol	Harvest Month × Washing	1, 19	5,10	0.0359
Ligstroside aglycone	Harvest Month	1, 28	9,14	0.0053
Naringenin	Harvest Month	1, 28	4,40	0.0451
Naringenin	Harvest Month × Washing	1, 19	4,38	0.0499
Oleocanthal	Harvest Month	1, 28	16,33	0.00038
Oleomissional	Bottling delay	2, 28	4,60	0.0187
Oleuropein aglycone	Harvest Month	1, 28	4,50	0.0429
p-Coumaric acid	Harvest Month	1, 28	9,13	0.0053
Syringaresinol	Destoning × Bottling delay	2, 19	4,50	0.0252
Total phenolics	Harvest Month	1, 61	6,51	0.0132
Total phenolics	Washing × Bottling delay	2, 52	3,56	0.0355
Tyrosol	Harvest Month × Bottling delay	2, 19	5,28	0.0150

Table S3. Validation metrics of the PLS-DA model used to discriminate olive oils according to harvest month. Model performance was evaluated using stratified 5-fold cross-validation. The optimal number of latent variables was selected based on the maximum Q^2 value, and model robustness was assessed using permutation testing (n = 500).

Factor	R^2X	R^2Y	Q^2	CV Accuracy (%)	Latent Variables	Cross-Validation	Permutations	Permutation p-value
Harvest month	0.47	0.92	0.64	96.9	3	Stratified 5-fold	500	0.002
